# A retrospective study showing maintenance treatment options for paediatric CD in the first year following diagnosis after induction of remission with EEN: supplemental enteral nutrition is better than nothing!

**DOI:** 10.1186/1471-230X-14-50

**Published:** 2014-03-20

**Authors:** Hazel Duncan, Elaine Buchanan, Tracey Cardigan, Vikki Garrick, Lee Curtis, Paraic McGrogan, Andrew Barclay, Richard K Russell

**Affiliations:** 1 Department of Nutrition & Dietetics, Yorkhill Hospital Glasgow, Dalnair Street, Glasgow G3 8SJ, United Kingdom; 2 Paediatric Gastroenterology Department, Yorkhill Hospital Glasgow, Glasgow, United Kingdom

**Keywords:** Crohn’s disease, Maintenance, Enteral nutrition, Children

## Abstract

**Background:**

A limited body of research suggests that ongoing maintenance enteral nutrition (MEN) can be beneficial in maintaining disease remission in Crohn’s Disease (CD). We aimed to assess how achievable MEN is and whether it helps to prolong remission.

**Methods:**

Patients newly diagnosed with CD in 2010 and 2011 who commenced exclusive enteral nutrition (EEN) for 8 weeks were followed up for a year post diagnosis. All patients who took EEN were encouraged to continue MEN post EEN. Data on azathioprine use was also collected. Categorical variables were compared using chi–square/Fischer’s exact test. Medians were expressed along with complete data ranges.

**Results:**

59 patients (34 male, median age 11.07 years, range 2.5-16.33 years) were identified. 11/59 (18%) had a poor response to EEN and were switched to steroids. 48/59 patients completed 8 weeks EEN and achieved clinical remission/response. 46/48 patients received Modulen IBD^®^, 29/48 (60%) consumed EEN orally and 19/48 (40%) via NGT. 15/48 (31%) patients were able to continue MEN post EEN completion. MEN was consumed for a mean of 10.8 months (range 4–14 months). 14/15 patients drank MEN and 1/15 had MEN via NGT. Remission rates at 1 year in patients continuing MEN were 60% (9/15) compared to15% (2/13) in patients taking no treatment (p = 0.001) and 65% (13/20) in patients taking azathioprine (p = 0.14).

**Conclusion:**

A sub group of patients can continue MEN as a maintenance treatment and this seems a useful strategy, especially in those who are not commencing azathioprine.

## Background

Crohn’s disease (CD) is characterised by patchy, transmural inflammation, which may affect any part of the gastro-intestinal tract from the mouth to the anus. It is a lifelong condition with periods of remission or relapse. The incidence of paediatric CD is rising, with a mean age at diagnosis in childhood of 11.9 years [1]. Presentation in the paediatric population is often with a variety of symptoms, abdominal pain is usually a prominent feature alongside persistent or recurrent diarrhoea with or without blood. Other symptoms include; nocturnal stooling, tenesmus, lethargy, anorexia and nausea as well as growth delay, delayed puberty and malnutrition [2]. Due to the diverse range of symptoms diagnosis can often be delayed, with associated issues of poor growth and undernutrition for all patients as well as delayed puberty in the adolescent patient group.

The aims of treatment include relief of symptoms, resumption of normal growth and pubertal development, prevention of relapse and complications as well as improvement in quality of life. The benefits of treatment need to be balanced against potential side effects. Nutritional therapy was first introduced as a treatment for CD in the 1970’s, [3] and since then EEN has been recognised in many centres as a first line treatment for active paediatric CD [4-7]. EEN has been shown to improve nutritional complications at diagnosis including weight loss, improving inflammatory markers and promoting mucosal healing [8].

Heuschkel et al. (2000) demonstrated that EEN was as effective as corticosteroids at inducing remission in children with CD, a finding replicated by an updated meta-analysis in 2007 [7,8]. EEN also has the benefit of having minimal side effects. Zachos et al. (2007) identified that while corticosteriods are commonly used in the treatment of adults with CD, their use in children has been met with caution due to their adverse effects notably on growth and bone mineral density [9].

After induction of remission in CD a strategic therapy will be chosen for maintenance of remission; most commonly, in the paediatric setting, azathioprine which carries potential toxicity risks and requires significant monitoring [10]. It is now felt that the role of EN may not just be limited to inducing remission but can also be used to maintain remission [11]. Nutritional support is key in maintaining normal growth and decreasing disease complications with minimal side effects from medications. There has however been a limited amount of research undertaken relating to EN as a maintenance treatment for patients with CD with many of the studies having been in adults [12]. The Cochrane report on Enteral Nutrition for Maintenance of Remission in CD only identified two papers which met their criteria for inclusion [13].

This study aimed to assess if MEN post induction of remission with EEN is achievable and if it helps prolong remission up to a year after diagnosis compared to other treatment strategies.

## Methods

### Patients

This study was carried out in a tertiary centre with all patients attending a specialist paediatric IBD clinic. All patients were diagnosed with Crohn’s disease using standard diagnostic criteria [14,15]. Response to treatment was defined by using a physician’s global assessment as described in previous studies from our centre [5].

A prospectively maintained departmental database was used to identify all children who had been diagnosed with CD in 2010 and 2011. All of the patients with active luminal CD who were commenced on EEN aiming to complete 8 weeks of treatment were included in the study. Patients were treated with either Modulen IBD (Nestle, Croydon, UK), Frebini Energy (Fresenius Kabi, Runcorn, UK) which was used for a patient under 5 years of age or Neocate Advance (SHS Nutricia, Liverpool, UK) which was used for a patient with co-existent cow’s milk protein intolerance.

All patients who responded to and completed 8 weeks EEN orally were universally encouraged to continue a smaller volume of maintenance enteral nutrition to maintain/optimise nutritional status as well as potentially providing a role in prolonging disease remission. Patients were encouraged to take MEN orally however if a NGT was insitu for the EEN course then it was often more difficult for them to consider supplemental nutrition post EEN. Patients were asked to take a volume of approximately 25% of their original EEN volumes although there was variability in this amount based on individual patient factors.

### Data collection

Data was collected retrospectively from departmental notes which included; date of diagnosis, type, volume and route of EEN received, whether clinical remission was achieved following EEN and whether the patient continued to take MEN. Clinical response or remission was determined using the physician’s global assessment. For the patients who continued to take MEN the length of treatment was documented. For all patients the date of relapse was documented and where applicable the date commencing azathioprine was also documented. There were no patients requiring treatment with methotrexate or infliximab during the 1 year study period. Follow up data was collected at the end of EEN period and then 6 months and 1 year post diagnosis for all EEN patients, not just those who took MEN. Relapse was defined as needing a further course of EEN or steroids within the follow up period.

Categorical variables were compared using chi-square test or Fischer’s exact test as appropriate. Statistical significance was defined as p value below 0.05. As an audit of clinical practice we have clarified previously from our ethics committee that no formal ethical approval was needed for this type of study.

## Results

### Patient population

**Figure 1:**
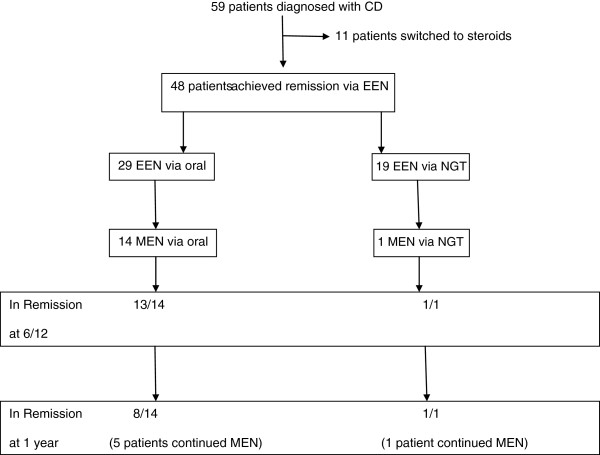
Patient overview and the overall clinical outcomes in 59 patients diagnosed with Crohn’s Disease split into those receiving EEN orally vs EEN via NGT.

Fifty-nine patients were diagnosed with CD over the two year period (Figure 1). Thirty-four of these patients were male. The median age at diagnosis was 11.07 years (range 2.5–16.33 years). Eleven (18%) patients were determined as having a poor response to EEN and were therefore switched to steroids during their initial EEN treatment course. No further follow up data was collected on patients switched to steroids. Twenty-nine (60%) of the patients received EEN orally whilst 19 (40%) received their feeds via NGT. Forty-six (96%) received Modulen IBD for their course of EEN with 1/48 receiving Frebini Energy and 1/48 receiving Neocate Advance. All patients had achieved clinical remission/response at the end of 8 weeks EEN.

Fifteen (31%) of patients who completed 8 weeks of EEN were able to continue MEN (11 male, 4 female). The median age of those continuing MEN was 12.08 years (range 2.5-14.9 years) compared to those unable to take MEN 11.75 years (range 2.5-15.91 years, p = ns). The MEN was taken for a median of 10 months (range 4–12 months). All of the patients who started MEN had taken EEN orally rather than by NGT except for the patient on Neocate Advance. The majority of patients (66%) drank Modulen IBD with 4 patients (26%) consuming Fortisip as MEN. The final patient received Neocate Advance via NGT aiming to provide almost full nutritional requirements due to the patient’s young age, a severe oral aversion and suspected multiple allergies which predated his diagnosis of IBD. There was no clear benefit for patients in remission compared with those who responded. Patients consumed varying quantities of MEN depending on age and appetite. Median volume of feed consumed was 400 ml (range 200-1000 ml) with a median energy intake of 400 calories (range 200–1000 calories) per day. We assessed the compliance and volume of feeds taken from dietetic, nursing and medical notes. Patients were offered telephone and email support throughout the treatment and therefore we hope to have optimised compliance however there was no specific objective way of validation.

Patients were split into 4 groups for analysis (see Table 1), those taking azathioprine & MEN, MEN only, azathioprine only and those on no active maintenance treatment (9, 6, 20 & 13 patients respectively). The number of patients who remained in remission from completing EEN and remained in remission at 6 months and 1 year was assessed. At 6 months follow up, patients on no treatment were significantly more likely to have relapsed compared to the other 3 groups (2/13 cf. 30/35 respectively, p = <0.001). Comparing patients specifically on MEN only to those on no treatment at 6 months patients on MEN were significantly more likely to remain in remission (6/6 cf. 2/13 respectively, p = 0.003).

**Table 1:** Remission rates for different maintenance treatment over the 1st year of follow up

**Treatment option**	**No. pts**	**Remission at 6 months**	**p-value cf. others at 6 months**	**Remission at 1 year**	**p-value cf. others at 1 year**
	**(n = 48)**				
MEN & azathioprine	9	8/9 (89%)	0.23	6/9 (67%)	0.46
MEN no azathioprine	6	6/6 (100%)	0.16	3/6 (50%)	0.66
No MEN, no azathioprine	13	2/13 (15%)	<0.001	2/13 (15%)	0.001
Azathioprine only	20	16/20 (80%)	0.18	13/20 (65%)	0.14

The remission rate in the MEN and azathioprine group was three times as many as those in the no treatment group (Table 1). Supplements were consumed for a mean of 10.8 months but with only 33% of patients still continuing to take MEN at time of relapse. Comparing all patients on MEN with those on no MEN, patients undertaking MEN were significantly more likely to be in remission at 6 months but not at 1 year (MEN 14/15 cf. no MEN 18/33, p = 0.02 at 6 months, MEN 9/15 cf. no MEN 15/33, p = 0.53 at 1 year).

## Discussion

This study summarises the use of MEN in paediatric CD patients from a complete treatment cohort who have all received EEN for induction of remission. The use of MEN at follow up was then compared alongside the use of azathioprine. The results from this study demonstrate that MEN is a useful strategy to help maintain remission. MEN is a favourable option especially in patients not taking azathioprine, but it’s uses are limited due to issues of palatability and ‘taste fatigue’ in a proportion of patients. Compliance can also be an issue potentially discouraging patients from taking future courses of EEN, however there is no specific research which has addressed this.

For many years immunosuppression has been used to maintain remission in the majority of patients with CD [16,17]. However these drugs have a recognised side effect profile [18]. This study provides further support for the use of nutritional therapy in children with CD both as an induction therapy but more specifically as a maintenance therapy. It has been well documented that EEN is an effective and favourable treatment option for patients diagnosed with CD, however until now there has only been a small amount of research looking at MEN [5,6,19].

Hania et al. (2012) have conducted the only randomised controlled trial comparing mercaptopurine (MP) and elemental diet as maintenance treatment for CD [20]. They studied 95 Japanese adult CD patients who were in clinical remission who were split into 3 maintenance groups (one group received MP, the second elemental diet and the third no active treatment). Follow up at 2 years demonstrated significantly higher remission in both patients receiving MP (56.7%) and patients receiving elemental diet (46.9%) compared to the control group (21.2%). Clearly this study has produced very similar results to the current study. Of note Hanai et al. (2012) had a longer follow up period, studied adult patients and used an elemental rather than a polymeric feed. In the study only 2/32 (6%) patients self-inserted an NG tube to receive elemental feeds, the remaining patients took supplemental drinks orally. The high numbers of patients able to drink elemental diet for 2 years is not something we think would be achievable in our clinical practice.

A Cochrane review identified two randomised control trials of MEN which met the inclusion criteria [13] and were both adult studies [21,22]. The study by Verma et al. (2001) had a moderately small sample size (33 adult patients who were steroid dependent) with no details documented as to the route of obtaining remission [21]. Of these 19 patients took elemental supplements and 14 patients took polymeric supplements. The supplements provided 35-50% of patient’s pre-trial calorie intake. Patients were followed up at 1 year and failure was determined as increase in Crohn’s disease activity index (CDAI), inability to withdraw steroids or surgery being required. 18% patients were unable to complete the study due to tolerance issues. 43% patients achieved and maintained remission and had steroids withdrawn. They identified similar remission rates in the polymeric diet group versus the elemental group. This study therefore demonstrates that whole protein sip feeds, which are generally more palatable, were effective at maintaining remission within this patient group.

The more recent study by Takagi et al. (2006) had a larger sample size of 51 and was carried out across two centres [22]. Patients were randomly assigned to 2 groups, the first group (n = 26) received half their nutritional requirements as an elemental diet and the second group (n = 25) had a free unrestricted diet with no nutritional supplementation. Remission had initially been achieved via surgery, 6–8 weeks EEN, IV methyl prednisolone or Infliximab. All patients were commenced on mesalazine and patients already on azathioprine prior to commencing study remained on the medication. They used an elemental diet providing 900-1200 ml and calories daily. 19% used an NG tube during the study period to obtain the elemental diet. 35% patients in the elemental diet group had relapsed by 2 years, compared to 64% in free diet group. While these studies are on adult patients using elemental feed the results are similar to our own.

This study demonstrates similar remission rates to other studies using EEN as an induction treatment at diagnosis of CD. A moderately high proportion of the patients received the EEN via NGT but this rate was similar to previous work by our group [5,23] as well as others [24]. Our centre has a strong multidisciplinary team (MDT) approach to patients undertaking EEN, with patients following a formalised pathway with regular MDT support. A full clinical review is carried out at 4 and 8 weeks. This has been shown to improve our numbers of patient able to complete 8 week courses of EEN [25]. Currently patients within our population group only managed to progress to MEN if they received EEN orally. Patients undertaking NG feeds for EEN were unable to consume MEN orally. However, patients were offered the same nutritional formulation for MEN as they were given for EEN which raises questions as to whether an alternative, potentially more palatable nutritional drinks for the individual patients could be used for maintenance. As such we now offer patients who have taken EEN by NG tube an alternative type of supplemental feed to try for maintenance therapy. We did not adopt an approach similar to Wilschanski et al. (1996) where they continued NG nutritional support as we felt this would be too difficult for patients to undertake [11]. However with this study and the other supportive data this may now be an option worth further exploration in selected patients.

There have been a small number of MEN studies within the paediatric population however these are non-randomised, non-blinded and all retrospective (Table 2). In our patient population 15/48 (31%) were able to continue MEN post achieving remission via EEN. These figures were lower than those identified by Knight et al. (2005) [26]. The number of patients able to continue MEN in our study was comparable to Day et al. (2006) who stated 31% of their patients continued maintenance supplemental drinks [27]. The feeds used in their study varied from elemental feeds to whole protein feeds and this is similar to feed choice in other studies. Other studies however do not define why certain feed choices were made [11,21,26].

**Table 2:** Summary of key papers in patients given MEN for maintenance of remission

**Paper**	**Total sample size**	**Number continued MEN**	**Route of supplementation**	**Type of supplementation**	**Volume taken**	**Remission rates at 1 year**
Verma et al. [21]	33	33	Oral	19 elemental	35-50% EAR	8/19 elemental
Adult population				14 polymeric		6/14 polymeric
Tageki et al. [22]	51	26	Oral/NG	Elemental	900-1200 ml/calories daily	9/29 elemental*
Adult population						
Wilschanski et al. [11]	47	28	NGT	elemental or semi-elemental feeds	50-60% EAR for 4 or 5 nights per week overnight	17/28 (p < 0.02)
Paediatric population						
Knight et al. [26]	40	22	Oral	Elemental or polymeric	1 litre daily	9/22 (not statistically sig)
Paediatric population						
Day et al. [27]	27	4	Oral	polymeric formula	300-1800 ml daily	4/4
Paediatric population						
Hanai et al. [20]	95	32	Oral/NG	elemental	>900 calories daily	20/32 (62.5%)
Adults						
Duncan et al.	48	15	Oral/NG	Polymeric or elemental	240-1000 ml	9/15
Paediatric population						

*Relapse rates at 1.5 years.

Contrary to the findings of our study and other published work, Knight et al. (2005) reported that MEN was not associated with a decreased relapse rate with no significant difference found between the group receiving MEN and the control group [26]. Their study focused on paediatric patients and the volumes of feeds to be taken were large at 1000 ml. This was felt to lead to issues with compliance and they documented that volumes consumed were variable.

In our study, the number of patients in remission at 6 months was statistically significant, however at 1 year was not. This could be due to patients stopping supplements due to compliance and taste fatigue. Following on from these results as a centre we feel it is important that support is optimised and a defined patient pathway would be beneficial to enhance compliance rates with MEN; our clinical practice has changed accordingly.

All studies (Table 2) state the volume of supplemental feeds consumed by patients; however it remains unclear from the literature whether this directly affects remission rates when using MEN. Early satiety and taste fatigue can be problematic if volumes are too high, however it is important that volumes are adequate to promote treatment efficacy. Taste fatigue is a commonly reported phenomenon in patients with chronic disease who take oral supplements [28]. This will limit the length of time supplements can be taken for; methods to minimise taste fatigue include looking at polymeric supplements which are often reported to be more palatable [29].

The mechanism of action of EEN or MEN is not fully understood. Several studies have shown that the gut microbiota is changed significantly after a course of EEN and once diet is introduced the gut flora reverts back to a similar state as pre EEN [30,31]. This raises the question-does MEN maintain the changes to gut microbiota and therefore maintain remission rates for longer? The studies examining this are small but do support the broad concept that MEN does seem to prolong a favourable bacterial profile compared to standard diet.

There are limitations to our study; sample size is moderate and all patients are from a single centre which has a strong track record for supporting patients undergoing EEN. The study is retrospective and non-randomised but has achieved 100% patient ascertainment and follow up over a 2 year period of all new diagnoses of CD. The follow up time is relatively short but with a significant fall off in patients managing to take supplements by this time is likely to be adequate for analysis purposes for this type of study.

## Conclusion

In conclusion, this study demonstrates that a sub group of patients can continue nutritional supplements post induction of remission with EEN as a maintenance treatment. This seems a useful strategy in a sub group of patients especially in those who are not commencing azathioprine. To our knowledge this is the first review evaluating MEN and azathioprine as an ongoing treatment option for this group in paediatrics. A randomised controlled trial would be beneficial in helping to clarify these provisional findings further.

## Competing interests

Dr Richard K Russell has received speaker’s fees, travel support, and participated in medical board meetings with MSD Immunology, Abbott, Dr Falk, Nestle and Ferring Pharmaceuticals. Dr Paraic McGrogan has received speaker fees, travel support and participated in medical board meetings with Nestle and MSD. V Garrick has received speaker’s fees, travel support, or participated in medical board meetings MSD, Ferring, Dr Falk & Nestle. E Buchanan has received speaker’s fees from Nestle. H Duncan has received speaker’s fees from Ferring.

## Authors’ contributions

HD and RKR prepared the manuscript with comments and corrections from all the authors. HD, TC, EB, VG, LC, PM, and AB helped collect the patient data. RKR/HD carried out the statistical analysis. All authors have read and approved the final draft. RKR acts as guarantor for the article.

## Pre-publication history

The pre-publication history for this paper can be accessed here:

http://www.biomedcentral.com/1471-230X/14/50/prepub
